# SARS-CoV-2 Natural Transmission from Human to Cat, Belgium, March 2020

**DOI:** 10.3201/eid2612.202223

**Published:** 2020-12

**Authors:** Mutien Garigliany, Anne-Sophie Van Laere, Cécile Clercx, Didier Giet, Nicolas Escriou, Christèle Huon, Sylvie van der Werf, Marc Eloit, Daniel Desmecht

**Affiliations:** University of Liège, Liège, Belgium (M. Garigliany, A.-S. Van Laere, C. Clercx, D. Giet, D. Desmecht);; Institut Pasteur, Paris, France (N. Escriou, C. Huon, S. van der Werf, M. Eloit);; Alfort National Veterinary School, Maisons Alfort, France (M. Eloit)

**Keywords:** 2019 novel coronavirus disease, coronavirus disease, COVID-19, severe acute respiratory syndrome coronavirus 2, SARS-CoV-2, viruses, respiratory infections, zoonoses, cat, *Felis silvestris catus*, reverse zoonosis, transmission, pandemic

## Abstract

In March 2020, a severe respiratory syndrome developed in a cat, 1 week after its owner received positive test results for severe acute respiratory syndrome coronavirus 2. Viral RNA was detected in the cat’s nasopharyngeal swab samples and vomitus or feces; immunoglobulin against the virus was found in convalescent-phase serum. Human-to-cat transmission is suspected.

We report the investigation of illness and infection with severe acute respiratory syndrome coronavirus 2 (SARS-CoV-2) in a household cat in Belgium (*1*). The cat was a female domestic shorthair, »15 years of age, that had been adopted 2 years earlier. The owner considered the cat to have been healthy since adoption, although it had never been assessed by a veterinarian. In February 2020, the owner took part in a 7-day tour to a mountain resort in Lombardy, Italy. The day after returning home, March 2, the owner felt suddenly too short of breath to conduct normal activities. As a precautionary measure, the family doctor decided to take a deep oropharyngeal swab sample and asked the patient to remain at home until the test result was reported. Over the next 10 days, the patient experienced a series of general, respiratory, and then digestive symptoms consistent with the clinical signs associated with coronavirus disease (COVID-19) (Figure). On March 6, the swab sample was declared positive for the SARS-CoV-2 genome, and home quarantine was extended until the end of March.

**Figure 7:**
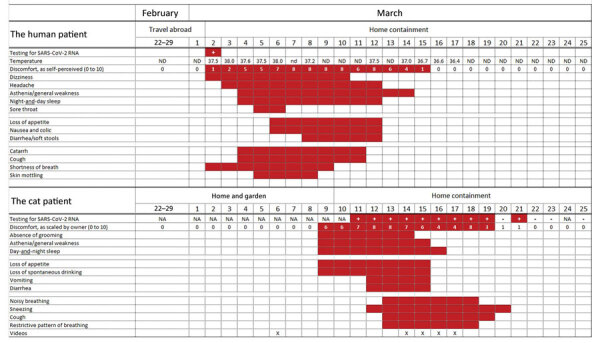
Timeline of disease course for human and cat with SARS-CoV-2 infection, by days from illness onset according to the cat owner, Belgium, February 22–March 25, 2020. NA, not available; ND, not determined; SARS-CoV-2, severe acute respiratory syndrome coronavirus 2.

During that time, the patient’s household cat was asymptomatic (Video 1). However, 1 week later, the cat suddenly demonstrated clinical signs; the cat was found prostrated and vomiting in her litter, then showed pronounced lethargy, poor appetite to anorexia, vomiting, and diarrhea (Figure). Several days later, the clinical signs worsened. The cat demonstrated sneezing (Video 2; Video 3); a harsh, productive cough several times a day; episodes of paroxysmal reverse sneezing (Video 4; Video 5); labored breathing with increased respiratory effort and frequency; and emaciation (Video 6). The clinical impression at this time was that of a restrictive breathing pattern suggestive of substantial involvement of parenchyma, pleura, or both. The cat’s condition then gradually improved; she became less lethargic, vomiting stopped, feces resumed normal consistency, episodes of cough became less frequent, and appetite quickly improved. The cat recovered completely within <2 weeks.

**Video 1:** Cat showing healthy behavior (March 6, 2020). **Video forthcoming**

**Video 2:** Cat showing reverse sneezing, suggestive of the presence of mucous or mucopurulent material in the throat/nasopharyngeal area (March 16, 2020).** Video forthcoming**

**Video 3:** Close-up of cat’s face showing open-mouth breathing, suggesting severe dyspnea, **Video forthcoming** nasal/nasopharyngeal obstruction, or both. The cat appears to be exhausted (March 15, 2020).

**Video 4:** Cat showing productive cough with terminal retch (March 14, 2020).** Video forthcoming**

**Video 5:** Cat showing productive cough with terminal retch (March 16, 2020).** Video forthcoming**

**Video 6:** Dry and deep cough with terminal retch and restrictive breathing pattern, suggestive of substantial parenchymal involvement. Cat is emaciated (March 17, 2020).** Video forthcoming**

A series of laboratory analyses were then conducted (Appendix). The cat’s owner collected 26 swab samples according to instructions received by telephone; 16 samples contained varying amounts of the SARS-CoV-2 genome (Table). Overall, positive samples were detected March 11–24. The cat was examined by veterinarians at the time of blood sampling on day 22 after onset of first symptoms. Clinical examination of the cat was unremarkable at that time, and auscultation of the thorax revealed no abnormalities. Results of a complete blood count and a serum biochemistry panel were within reference ranges. Presence of serum IgG was first sought by Western blotting of mock-exposed and SARS-CoV-2–exposed Vero E6 cells lysates. In convalescent-phase serum, 5 protein bands that were simultaneously absent from mock-exposed Vero E6 cell lysates were identified (Appendix Figure). Furthermore, the convalescent-phase serum was positive by double-epitope sandwich ELISA and for 2 of the 3 antigens tested by double-epitope luciferase assay (Table; Appendix Table). Whereas serum samples from 30 control cats and 10 control humans were negative by virus neutralization assay, the convalescent-phase serum samples from the cat and her owner were positive; endpoints were 1:512 for the cat and 1:128 for the human.

**Table:** Severe acute respiratory syndrome coronavirus 2 genome loads measured by qRT-PCR in a series of consecutive swab samples from cat, Belgium, March 2020*

Date	Oropharyngeal swab samples			Vomitus			Feces	
	β-actin gene	N gene		β−actin gene	N gene		β-actin gene	N gene
11	NS	NS		26.95	23.5 ± 0.1		35.5	33.3 ± 0.2
12	33.4	38.2 ± 0.5		NS	NS		32.9	34.8 ± 0.0
13	37.8	37.9 ± 0		ND	34.9 ± 0.1		34.4	37,6 ± 0,1
14	25.1	39.3 ± 0.1		NS	NS		30.7	35.1 ± 0.1
15	35.9	35.7 ± 0.1		NS	NS		27.8	33.2 ± 0.1
16	38.2	Negative		NS	NS		26.0	35.1 ± 0
17	27.1	38.2 ± 0		NS	NS		28.7	Negative
18	26.7	Negative		NS	NS		36.1	37.9 ± 0
19	NS	NS		NS	NS		27.9	39.0 ± 0
20	NS	NS		NS	NS		30.1	Negative
21	NS	NS		29.9	33.8 ± 0.1		32.8	Negative
22	37.9	Negative		NS	NS		31.7	Negative
23	NS	NS		NS	NS		33.8	Negative
25	NS	NS		34.9	Negative		35.0	Negative
*Numbers reported are defined as the number of cycles required for the real-time PCR assay fluorescent signal curve to intersect with a threshold line that exceeds background level (mean ± SD). It is a relative measure of the concentration of the genomic target in the qRT-PCR reaction (the severe acute respiratory syndrome coronavirus 2 N gene or cat β-actin gene); values >40 are considered negative. All samples with qRT-PCR values <40 were analyzed further by a standard gel RT-PCR targeting the coding sequence of the virus spike protein gene followed by Sanger sequencing of the correctly sized amplicon retrieved (»370 bp). Only samples positive for all 3 tests were defined as positive, which was the case for all samples with a value <40 for the N gene aggregated in this table. NS, no sample available; negative, RT-qPCR and/or gel PCR and/or sequencing test failed; qRT-PCR, quantitative reverse transcription PCR.								

The cat at first showed general signs, then gastrointestinal signs, and finally respiratory signs, similar to those observed in humans. Subsequently examined samples from the cat revealed viral RNA persisting for about 10 days. With the exception of a vomitus fluid sample collected on March 13, the amounts of viral RNA were relatively low. For this reason, and despite the simultaneous presence of a compatible clinical syndrome and a suggestive chronology of events, we cannot automatically rule out passive contamination of the cat’s samples by its owner.

To confirm the hypothesis of a productive infection of the cat, we conducted a series of serologic analyses by using 4 different testing approaches and targeting distinct viral protein targets. All procedures converged toward the same result: the convalescent-phase serum from the cat contained immunoglobulins against SARS-CoV-2, which were absent from the serum from control cats. These antibodies target several distinct viral proteins, and they caused a total neutralizing effect up to a much higher dilution than those from the owner’s serum. This household cat was therefore productively infected with the SARS-CoV-2 virus excreted by its owner, and the infection caused a nonfatal but nevertheless severe disease, mainly of the respiratory system (Videos 2–6).

Public health officials are still learning about SARS-CoV-2, but no current evidence indicates that pets play a role in spreading the virus. Therefore, taking measures against companion animals that may compromise their welfare is not justified.

Appendix Additional materials and methods for study of natural transmission of severe acute respiratory syndrome coronavirus 2 infection from human to cat, Belgium, March 2020.
