# Supplementary material for: SARS-CoV-2 Natural Transmission from Human to Cat, Belgium, March 2020
Source: Emerg Infect Dis. 2020 Dec;26(12):3069–71. doi: 10.3201/eid2612.202223 (PMC7706966; doi:10.3201/eid2612.202223)
Supplement: Appendix — Additional materials and methods for study of natural transmission of severe acute respiratory syndrome coronavirus 2 infection from human to cat, Belgium, March 2020. [file 20-2223-Techapp-s1.pdf]

Article DOI: https://doi.org/10.3201/eid2612.202223

SARS-CoV-2 Natural Transmission from Human to Cat, Belgium, March 2020

Appendix

Materials & Methods related to the case study

Clinical data

Due to the strict confinement imposed to the owner, it would have been inconsistent to ask a veterinarian to enter the house to conduct a clinical examination of the animal and to collect samples. The clinical signs were therefore aggregated indirectly by questioning the owner on the phone and by visualizing the daily videos she was asked to send. For the samples, instructions were given to perform deep oro-pharyngeal mucosal swabs, vomit swabs and faeces swabs. The swabs were then stored at 4°C in a tube for up to 24 hours and deposited on the front door step of the house where an attendant collected them every morning. On March 31, seven days after the last positive SARS-CoV-2 sample, a jugular blood sample was collected under general anaesthesia, centrifuged and the corresponding serum stored at -20°C until serological testing was performed.

Virological diagnosis

The presence of the SARS-CoV-2 genome was first ascertained by reverse transcriptase quantitative PCR (RT-qPCR) using the β-actin transcript as internal control. The primer-probe set was selected based on sequence information from the Centers for Disease Control and Prevention (USA, reagent label 2019-nCoV_N1). Each swab was immersed and agitated in 500 µL of PBS for 30 sec. and the RNA was isolated using the Nucleo Spin RNA® Set for NucleoZOL according to the manufacturer's instructions (Macherey Nagel). RT-qPCR detection and quantitation of target viral N1 sequence was made using the Luna® Universal Probe qPCR Master Mix (New England Biolabs) and the StepOnePlus real time PCR instrument (ThermoFisher). Cycle threshold (Ct) values above 40 were considered negative. Samples proved positive twice by RT-qPCR were then confirmed further by a standard gel RT-PCR targeting the RBS coding sequence of the spike protein gene (using in-house primers RBD-F 5′-GCAAACTGGAAAGATTGCTGA-3′ and RBD-R 5′-ACCAAATTAGTAGACTTTTTAGGTCCA-3′) followed by subsequent Sanger’s sequencing of

the correctly sized amplicon retrieved (≈370 bp). Only samples positive for all three tests were considered positive.

Serological diagnosis

Four methods were used to detect possible anti-SARS-CoV-2 antibodies. Three months after the pandemic emergence, no serodiagnostic tool specifically validated for the feline species was available. In this context, we opted for the use of very different tools, implemented by 3 independent laboratories, and targeting different viral targets. The cat sample was anonymised and aggregated with 1-5 reference samples taken before December 2019 to allow blind testing.

Immunoblotting — The SARS-CoV-2 isolate Belgium/Sart Tilman/2020/01 was derived from a positively-testing nasopharyngeal swab in the University Hospital. Infection of Vero E6 cells was carried out in phosphate-buffered saline (PBS) containing 2% fetal calf serum. The inoculum was added to the cells for 1 h at 37°C, after which cells were washed twice with PBS and maintained in Dulbecco's Modified Eagle's Medium (Lonza) with 2% FCS, 2mM glutamine and antibiotics for 48 h. Protein extracts (PE) from SARS-CoV-2-infected and uninfected Vero E6 cell monolayers were prepared in RIPA buffer. Protein extract (9 µg), NuPAGE LDS Sample Buffer (6,25 µL) and reducing agent (2,5 µL) were mixed, heated at 70°C for 10 min and, along with a protein ladder (5 µL, Precision Plus Protein™ Dual Xtra Prestained Protein Standards, Biorad), loaded in a 4-12% Bis-Tris Protein Gel. Gels were run at 200 V for 1 h using the XCell SureLock™ mini-cell electrophoresis system. Migrated proteins were transferred onto polyvinylidene difluoride membranes, nonspecific binding sites were blocked with skim milk (5% in PBST) for 2 h at RT and membranes were probed overnight at 4°C with feline or human serum diluted 1/100 in PBST/skim milk (2,5%). Blots were washed with PBST and incubated with an HRP-conjugated goat anti-feline (ab112801, from Abcam) or anti-human (ab97225, from Abcam) IgGs antibody (diluted 1/10000 v/v in PBS-Tween/skim milk) for 1 h at RT. Membranes were washed 3 times with PBST on a plate shaker for 5 min, then in deionized water for 2 min. Membranes were revealed using the chemiluminescent substrate Novex® ECL (Invitrogen) according to the manufacturer's protocol. Visualisation was made using the imaging system ImageQuant™ LAS 4000 and ImageJ software (3 min exposition). After, membranes were washed in PBST, incubated 1 h at RT with an HRP-conjugated anti-b-actin antibody (ab49900, from Abcam) diluted 1/25000 v/v in PBST/skim milk, then revealed and vizualised as described.

Double-epitope sandwich enzyme immunoassay — Platelia™ SARS-CoV-2 Total Ab is a one-step assay for the semi-quantitative detection of antibodies IgM, IgA and IgG to the

SARS-CoV-2 nucleocapsid in human serum using EIA technology (ref. #72710, from Bio-Rad). Briefly, the test uses a nucleocapsid protein in two forms: either coated on the bottom of the wells or conjugated to horseradish peroxidase. The conjugate is added to the serum sample and the mixture is then incubated for one hour at 37°C in the sensitised well. During this incubation step, if IgM and/or IgG and/or IgA antibodies are present in the sample, an immune complex is formed by aggregation of the recombinant nucleocapsid proteins deposited on the surface of the well, the specific antibodies and the recombinant nucleocapsid proteins coupled to peroxidase. The presence of the immune complexes is then demonstrated in a conventional manner by the distribution of a chromogenic solution inducing a colour reaction read with the spectrophotometer at 450/620 nm ; the presence of specific antibodies in a sample being demonstrated by performing a ratio between the optical density of the sample and that of the threshold control. This tool had been initially developed for the detection of anti-SARS-CoV-1 antibodies in human serum/plasma and it proved insensitive to antibody cross-reactions against seasonal human coronaviruses. It appears that this assay very reliably detects the serological response to SARS-CoV-2 infections, presumably due to the phylogenetic proximity between the two nucleoproteins. Further, we hypothesized that the format of this technology, in which specific ligands are sandwiched between two copies of their target, should in principle work to detect specific antibodies produced by other species.

Luciferase immunoprecipitation assay — Schematically, this assay is based on the use of recombinant NanoLuc (Nluc) luciferase-antigen fusions (*1*,*2*). The assay is initiated by incubating crude Nluc-antigen containing cell extracts with patient/animal sera in microtiter wells. The immune complexes are then precipitated onto a filter plate by protein A/G-coated beads. After washing, antibody-bound Nluc-antigen is measured by the addition of coelenterazine substrate and light units are measured with a luminometer. Three specific Nluc-antigen fusions were produced and validated by the Pasteur Institute for profiling antibody responses against SARS-CoV-2 : Nluc-full_S1_subunit_(residues 1-698), Nluc-full_S2_subunit_(686-1208) and Nluc-full_N_(1-419) (L. Grzelak et al., unpub. data, https://www.medrxiv.org/content/10.1101/2020.04.21.20068858v1). These novel reagents were used to test the cat sera.

Virus neutralization assay — Vero E6 cells were seeded in 96-well plates at 2.10^4 cells/well. The day after, 100 $TCID_{50}$ of virus (isolate Belgium/Sart Tilman/2020/01) were incubated with serial 2-fold dilutions of sera, starting from 1:10, in 100 µl of DMEM for 1 hour at 37°C. Mixes were then added to cells and incubated for 1 hour at 37°C. Virus/sera mixes were removed, 100µl of DMEM were added, and cells were incubated for 5 days at 37°C with 5%

CO_2. Virus inoculum was back titrated in each experiment. Neutralisation was assessed by CPE, the reading of which was performed by independent, blind direct observations under the inverted microscope and after cell coloration with crystal violet. All these operations were carried out in a level 3 biosafety laboratory. The neutralisation endpoint was determined as the serum highest dilution that inhibited 100% of the SARS-CoV-2 infection observed by CPE of inoculated cells.

References

1. Burbelo PD, Ching KH, Bush ER, Han BL, Iadarola MJ. Antibody-profiling technologies for studying humoral responses to infectious agents. Expert Rev Vaccines. 2010;9:567–78. 10.1586/erv.10.50 PubMed https://doi.org/10.1586/erv.10.50

2. Burbelo PD, Ching KH, Klimavicz CM, Iadarola MJ. Antibody profiling by luciferase immunoprecipitation systems (LIPS). J Vis Exp. 2009;7:1549. 10.3791/1549 PubMed https://doi.org/10.3791/1549

Appendix Table. Serologic data*

Testing tool/procedure	Cut-off value	Control cat serum	Case cat serum	Conclusion
Double-epitope sandwich ELISA	1,35	0,90	2,89	Positive
Luciferase immunoprecipitation				
S1 domain	0,52	0,02	1,29	Positive
S2 domain	1,20	0,21	7,07	Positive
C-term N protein	0,15	0,01	0,11	Dubious
Virus neutralization test	NA	<1:10	1:512	Positive

*Cut-off values have been established using a set of prepandemic and SARS-CoV-2-positive human sera. Values above the cut-off are considered positive. NA, not applicable. See text for detailed description of the different tools/procedures. Blood had been collected 3 weeks after first clinical signs appeared.

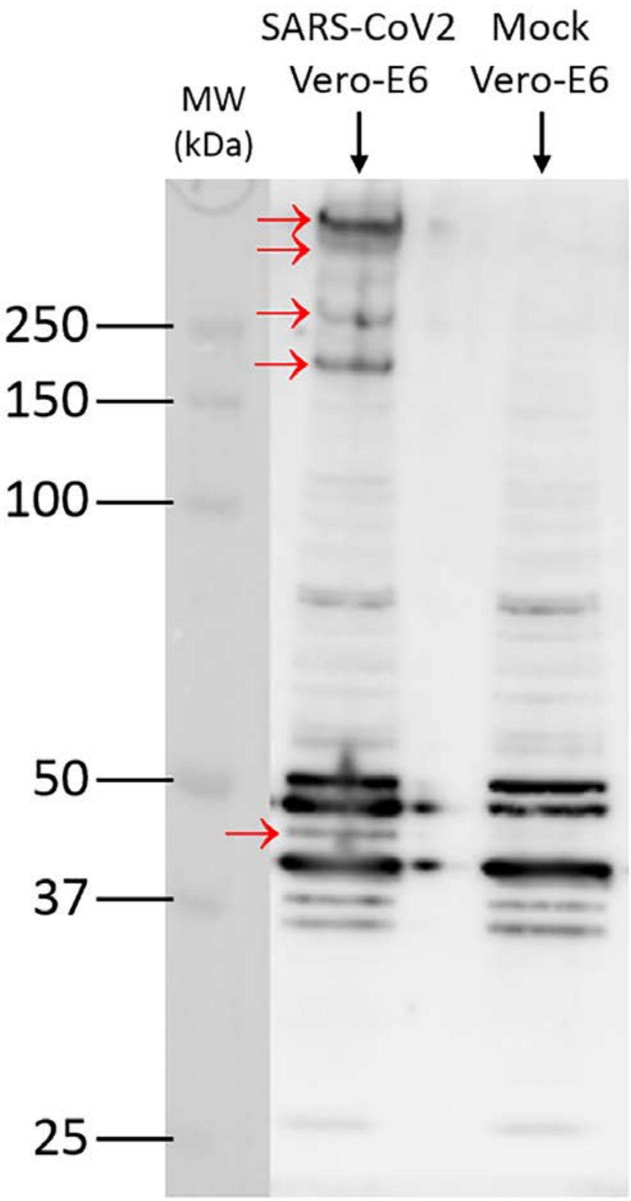

Appendix Figure. Western blot probing of mock- and SARS-CoV-2 infected Vero E6 cell lysates using cat serum. The strip was probed with convalescent cat serum diluted 1:100 and exposed for 3-min. The arrows indicate SARS-CoV-2 protein bands. The bands migrating at ca. 180 and 250 kDa probably represents two glycoforms of the spike and the band at ca. 45 kDa the nucleoprotein. The heavy bands at ca. 700 and 800 kda could represent the replicase. Probing with control cat sera did not display these bands. MW, molecular weight.
